# Molecular characterization of extended-spectrum beta-lactamase-producing *Escherichia coli* among children and farm animals in Agogo, Ghana

**DOI:** 10.1186/s12866-026-04978-w

**Published:** 2026-03-25

**Authors:** Charity Wiafe Akenten, Hagen Frickmann, Thorsten Thye, Neyaz Ahmed Khan, Ralf Krumkamp, Ellis Kobina Paintsil, Linda Aurelia Ofori, Anna Jaeger, Wibke Loag, Maike Lamshoeft, Ernst Molitor, Achim Hoerauf, Kwasi Obiri-Danso, Stefan Berg, Oumou Maiga Ascofare, Richard Odame Phillips, Jürgen May, Denise Dekker

**Affiliations:** 1 Kumasi Centre for Collaborative Research in Tropical Medicine (KCCR), Kumasi, Ghana; 2 Department of Microbiology and Hospital Hygiene, Bundeswehr Hospital, Hamburg, Germany; 3 Institute for Medical Microbiology, Virology and Hygiene, University Medicine Rostock, Rostock, Germany; 4 Infectious Disease Epidemiology Department, Bernhard Nocht Institute for Tropical Medicine Hamburg, Hamburg, Germany; 5 Research Group One Health Bacteriology, Bernhard Nocht Institute for Tropical Medicine, Hamburg, Germany; 6 Department of Theoretical and Applied Biology, Kwame Nkrumah University of Science and Technology, Kumasi, Ghana; 7 Institute for Medical Microbiology, Immunology and Parasitology, University Hospital, Bonn, Germany; 8 German Centre for Infection Research, partner site Bonn-Cologne, Bonn, Germany; 9 University Medical Centre Hamburg-Eppendorf (UKE), Hamburg, Germany

**Keywords:** Enterobacterales, Antimicrobial resistance, *Escherichia coli*, Aarm animal, β-lactamases, Children, Whole-genome sequencing, Ghana

## Abstract

Enterobacterales such as *Escherichia coli* are known to produce extended-spectrum β-lactamase (ESBL) enzymes at high rates in Ghana, West Africa. However, little is known about the One Health connections that may facilitate the dissemination of these resistant bacteria. This study aimed to use whole-genome sequencing (WGS) to investigate resistance determinants, virulence genes, and associated mobile genetic elements (MGEs) of ESBL-producing *E. coli* isolated from stool samples of Ghanaian children under five years of age, both with and without diarrhoea, and from farm animals, including poultry and goats.

A total of 117 confirmed ESBL-positive *E. coli* isolates recovered from stool samples of children without diarrhoea (44/117), children with diarrhoea (30/117), poultry (35/117), and goats (8/117) were subjected to WGS. Phylogenetic analyses based on multilocus sequence typing (MLST) identified 49 distinct sequence types (STs), with ST617 and ST38 among the most prevalent lineages. ESBL production was predominantly mediated by the *bla*_CTX−M−15_ gene. Overlapping resistant genes and ST profiles observed between human and animal isolates suggest shared reservoirs. In summary, the work provides region-specific genomic evidence of shared sequence types, resistance genes, virulence factors, and mobile genetic elements among ESBL-producing *E. coli* isolates from children and farm animals in Ghana. These findings highlight the potential dissemination of multidrug-resistant strains and fill an important knowledge gap within a One Health context. Thus, the study provides a region-specific baseline for future molecular epidemiological surveillance and One Health–focused investigations in Ghana.

**Supplementary Information:**

The online version contains supplementary material available at 10.1186/s12866-026-04978-w.

## Introduction

Antimicrobial resistance (AMR) is a growing global health threat, with projections estimating up to 10 million deaths annually by 2050 if urgent action is not taken [1]. AMR *Escherichia coli* (*E. coli*) are among Ghana’s most frequently isolated Gram-negative bacteria from patients with urinary tract and bloodstream infections [2]. While *E. coli* commonly exists as a commensal organism in the intestinal microbiota of humans and animals, certain lineages can cause both intestinal and extraintestinal infections. Its ability to acquire and disseminate AMR across human, animal, and environmental reservoirs makes it a key organism of interest within the One Health framework [3]. Resistance to commonly used antibiotics in Ghanaian hospitals often leads to difficult-to-treat infections and is associated with poor clinical outcomes [4, 5].

AMR *E. coli* have been reported in Ghana for several decades [5], with a notable increase in resistance mediated by extended-spectrum beta-lactamases (ESBLs). ESBL-producing *E. coli* often exhibit multidrug resistance (MDR), including resistance to fluoroquinolones, aminoglycosides, and sulfonamides, further complicating treatment options [6].

Globally, CTX-M-type enzymes, particularly those encoded by the *bla*_CTX−M−15_ gene, are the most prevalent ESBL determinants in *E. coli* [7]. This resistance gene is highly transmissible due to its location on mobile genetic elements like plasmids, facilitating its dissemination across bacterial populations and ecological niches. In Ghana and sub-Saharan Africa, *bla*_CTX−M−15_ has been detected in human, animal, and environmental isolates, suggesting the presence of shared resistance reservoirs and potential circulation within One Health systems. Infections caused by *bla*_CTX−M−15−_producing bacteria are associated with increased risk of treatment failure, especially where antimicrobial susceptibility testing is limited and empiric therapy may not adequately cover resistant strains [7].

Several clonal lineages, ST10, ST617, and ST131 have been associated with ESBL-mediated resistance in Ghanaian patients and livestock [8–10], while other studies have reported substantial genetic diversity among resistant isolates [11]. In West Africa, STs such as ST617 and ST38 are commonly detected and often carry *bla*_CTX−M−15_. The presence of ESBL-producing *E. coli* in human [2], veterinary [12], and environmental sources [13] highlights the interconnected nature of AMR within a One Health context. However, despite increasing reports of ESBL-producing *E. coli* in Ghana, there remains limited genomic data comparing isolates from human and animal reservoirs, particularly in vulnerable populations such as young children.

To address this gap, this study applied WGS to characterize ESBL-producing *E. coli* isolates from stool samples of Ghanaian children under five years of age, both with and without diarrhoea, and from farm animals, including poultry and goats.

The study aimed to investigate resistance determinants, virulence genes, and associated mobile genetic elements (MGEs), and to assess genomic similarities between human and animal isolates. Improved understanding of the genomic epidemiology of ESBL-producing *E. coli* across human and animal reservoirs will help inform surveillance strategies and support efforts to mitigate the spread of antimicrobial resistance in Ghana.

## Materials and methods

### Study site, study population and sample collection

The isolates analysed in this study were obtained from our previously conducted cross-sectional study in Agogo, a peri-rural town in the Asante Akim North District within the Ashanti Region of Ghana, as described elsewhere [14, 15]. In the original study, stool samples were collected between July and December 2019 from children under 5 years of age, both with and without diarrhoea, attending healthcare facilities within the Agogo hospital catchment area, as well as from farm animals, including poultry and goats, located in the surrounding communities. Fresh faecal samples were collected using sterile spoons, avoiding ground contact, and transported at 2–8 °C to the Kumasi Centre for Collaborative Research in Tropical Medicine (KCCR) within 2–4 h for microbiological analysis, as previously reported [14, 15]. A total of 470 phenotypically confirmed ESBL-producing *E. coli* isolates were identified and stored during the original study. Farm participation was voluntary, and ethical approval was obtained from institutional review boards.

### Selection of isolates for whole genome sequencing

For the present study, out of 470 confirmed ESBL-producing isolates, 117 were randomly selected for WGS. Isolates eligible for inclusion had complete metadata and a confirmed ESBL phenotype. Random selection was performed using Microsoft Excel by assigning each isolate a unique identification number and generating random values using the RAND() function. The dataset was sorted by the generated random numbers, and isolates were selected proportionally within host groups to maintain representation consistent with their distribution in the main samples. The sample size was determined based on available WGS resources while ensuring adequate representation for One Health analysis. No additional selection based on antimicrobial resistance phenotype, sequence type, or clinical characteristics was performed, thereby minimizing potential selection bias.

### DNA extraction

Genomic DNA was extracted using the MasterPure Complete DNA and RNA Purification Kit (Lucigen Corp., now LGB Biosearch Technologies, USA). The Quant-iT dsDNA Assay Kit (Thermo Fisher Q33130, Germany) was used to determine DNA concentration.

### Sequencing platform

DNA samples were sent to BGI Tech Solutions (Hong Kong) for genome sequencing. Only samples with a volume of at least 20 µL, a concentration of ≥ 20 ng/µL, and an A260/280 ratio between 1.8 and 2.0 were submitted.

### Assembly and quality control

Assembled contigs were annotated using BAKTA v1.14.6 (SM1.5). The core genome was determined by applying PANAROO v3.13.0 (SM1.6) and acquiring GFF files with default settings. A core genome alignment was done with MAFFT v7.467 (SM1.7). The quality of raw sequencing data was checked with the AQUAMIS software v1.2.0, which implements FASTP v0.19.5 for trimming, SHOVILL v1.1.0 (SM1.1), and assembly quality analysis using QUAST v5.0.2 (SM1.2) (assemblies were retained if they < 300 contigs, and an assembly N50 ≥ 50 kb). In addition, contamination checks of the assembled bacterial genomes were done with CONFINDR v0.7.1 (SM1.3) and CHECKM2 (genomes passing quality control showed ≥ 99% completeness and < 1.5% contamination) (SM1.4). (See attached Supplementary materials_1 for all software)

### Multilocus sequence typing and phylogeny construction

MLST analysis was conducted with MLST Torsten Seeman v2.23.0 (SM1.14). Sequences that initially did not match any known STs were subsequently uploaded as FASTQ files to EnteroBase, resulting in the assignment of three novel STs (accession code: CRA023281). Based on the WGS data of all 117 samples, an in silico phylogroup analysis of *E. coli*, which comprises eight primary phylogroups (A, B1, B2, C, D, E, F, and G), was conducted using the Clermon Typing pipeline [16]. All 117 assembled *E. coli* genomes were analyzed at the whole-genome level using a maximum likelihood phylogenetic tree created with the SNIPPYSNAKE pipeline (SM1.8). The phylogenetic tree was constructed using IQ-TREE (SM1.9). Clonality was defined by 5 or fewer SNPs [17] differences between two isolates.

### Antimicrobial resistant gene detection

Antimicrobial resistance determinants, plasmid replicons, and virulence factors were identified in silico across all *E. coli* genome assemblies. Resistance genes were detected with the AMRFinderPlus v3.10 (SM1.10) software. (See attached Supplementary materials_1 for all software)

### Virulence gene analysis

The presence of *E. coli*-specific virulence genes was assessed with ABRICATE v1.0.1 (SM1.11), utilizing the *E. coli* _VF database (SM1.12). For this study, diarrhoeagenic *E. coli* (EAEC) was based on the presence of *aggR* together with associated EAEC virulence determinants. Therefore, isolates carrying *aatA* alone were not designated as EAEC [18].

### Mobile genetic element analysis

Plasmid screening was performed with the MOB-SUITE package (SM1.13) using the mob-cluster algorithm, and the Plasmidfinder database was screened with the ABRICATE software. (See attached Supplementary materials_1 for all software)

### Genotype–phenotype concordance analysis

Phenotypic antimicrobial susceptibility results were interpreted according to EUCAST clinical breakpoints (version 10.0, 2020). Genotypic resistance was defined as the presence of known antimicrobial resistance genes or resistance-associated mutations identified through WGS using established resistance gene databases. The relationship between phenotypic and genotypic resistance was assessed by comparing the presence or absence of resistance genes with the corresponding phenotypic susceptibility results for each antimicrobial agent. were recorded and reported descriptively.

## Results

### Phylogenetic structure, phylogroup distribution, and sequence type diversity of ESBL-producing *E. coli*

**Fig. 1:**
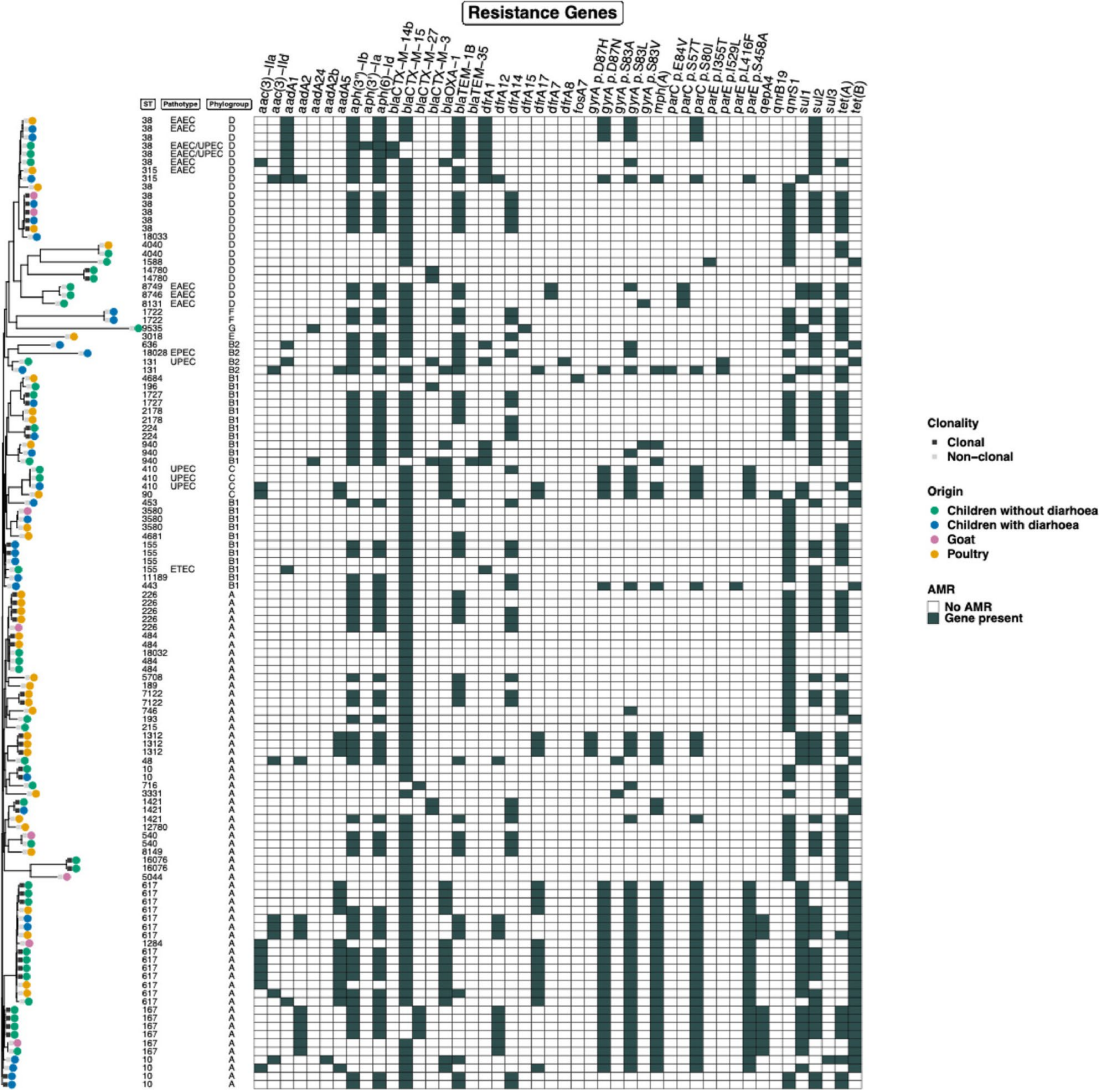
Phylogenetic tree constructed based on the MLST analysis of 117 *E. coli* genomes. The heatmap shows selected AMR gene classes, sequence types (STs), pathotypes, and plasmids. Only a subset of AMR genes is displayed for clarity. Clonality was defined as < 6 SNPs; clonal isolates are indicated by black squares with colored dots, and non-clonal isolates by grey squares with dots. The full dataset and additional details are provided in Supplementary Material (ST_host_resistance_SM)

Phylogenetic analysis of 117 ESBL-producing *E. coli* genomes demonstrated substantial genetic diversity, with isolates distributed across multiple branches of the phylogenetic tree rather than forming host-specific clusters (Fig. 1). Isolates from children with and without diarrhoea, poultry, and goats were interspersed throughout the tree, indicating diverse genetic lineages across host sources.

Phylogroup assignment showed that the majority of isolates belonged to phylogroup A, followed by phylogroups D and B1, with smaller proportions assigned to phylogroups B2, C, E, F, and G (Table 1). The dominant phylogroups included isolates from both human and animal origins.

**Table 1:** Distribution of ESBL-producing *E. coli* phylogroups across host sources

Phylogroup	Children with diarrhoea *n* (%)	Children without diarrhoea *n* (%)	Poultry *n* (%)	Goats *n* (%)	Total *n* (%)
A	8 (26.7)	25 (56.8)	22 (62.9)	5 (62.5)	60 (51.2)
B1	10 (33.3)	5 (11.4)	6 (17.1)	1 (12.5)	22 (18.8)
B2	3 (10.0)	1 (2.3)	0	0	4 (3.4)
C	1 (3.3)	2 (4.5)	1 (2.9)	0	4 (3.4)
D	6 (20.0)	10 (22.7)	5 (14.3)	2 (25.0)	23 (19.7)
E	0	0	1 (2.9)	0	1 (0.9)
F	2 (6.7 )	0	0	0	2 (1.7)
G	0	1(2.3)	0	0	1 (0.9)
Total	30	44	35	8	117

Multilocus sequence typing identified 49 distinct sequence types (STs), including three novel STs (ST18028, ST18032, and ST18033), reflecting substantial ST diversity. The four most frequent STs were ST617, ST38, ST10, and ST167 in a declining order of frequency. ST10 and ST617 in particular were predominantly associated with phylogroup A and were detected in isolates from children, poultry, and goats, whereas ST38 and ST167 were primarily linked to phylogroup D and were identified in both human and animal isolates. The globally disseminated lineage ST131 [19] was detected in two isolates from children. Overall, 25 STs were exclusive to child isolates and 14 to animal isolates, while 10 STs were shared across host groups. These shared STs were distributed within the dominant phylogroups A, B1, and D. Details are shown in Fig. 1; Table 1.

### Distribution of AMR genes

The most predominant AMR genes identified among the 117 successfully typed genomes included the ESBL gene *bla*_CTX−M−15,_ which was recorded in nearly 9 out of 10 isolates. followed by genes mediating resistance to aminoglycosides, sulphonamide, and quinolones. The average number of AMR genes per genome was similar across bacteria from children with diarrhoea (*n* = 14), children without diarrhoea (*n* = 13), poultry (*n* = 12), and goats (*n* = 12). In addition to the ESBL-type beta-lactamase gene *bla*_CTX−M−15_, genes *bla*_TEM−1_ and *bla*_OXA−1_, both associated with broad-spectrum beta-lactam resistance, were recorded in declining order of frequency. Several less frequently detected resistance genes such as tetracycline, fluoroquinolone etc. were found and are detailed in Fig. 1 and Supplementary Table S1.

### Comparison of genotypic and phenotypic resistance

A total of five antibiotic classes were analyzed to compare genotypic and phenotypic resistance (Table 2). The phenotypic susceptibility data were retrieved from previously published studies [14, 15]. All the 117 isolates carried β-lactam resistance genes and were phenotypically resistant, showing complete genotype–phenotype concordance. For other antibiotic classes, varying levels of discordance were observed, as shown in Table 2.

**Table 2:** Genotype–phenotype comparison of antimicrobial resistance (*N* = 117)

Antibiotic Class	Resistance Genes Detected	Isolates with AMR Genes (*n*)	Phenotypically Resistant Isolates (*n*)
β-Lactams (Penicillins & Cephalosporins)	*bla*_CTX−M−15_, *bla*_CTX−M−27_, *bla*_CTX−M−3_, *bla*_CTX−M−9_, *bla*_TEM−1_, *bla*_OXA−1_	117	117
Aminoglycosides	*aac(6’)-Ib-D181Y*,* aph(3’’)-Ib*,* aac(3)-Iie*	15	18
Fluoroquinolones	*qnrS1*,* gyrA*,* parC*	107	41
Sulfonamides	*sul1*,* sul2*,* sul3*	64	83
Tetracyclines	*tetA*,* tetB*,* tetD*	94	101

### Mobile genetic elements (Plasmid families)

Sixteen types of mobile genetic elements (MGEs) were identified among the isolates, with several appearing only once. *IncY* was the most common MGE, detected in 19 isolates across all host groups. This was followed by *IncFIB*, *Col(BS512)*, *IncFII*, and *rep_cluster_488* in declining order of frequency. Absolute numbers, distribution over the subpopulation, as well as some less frequently detected MGEs, are shown in Supplementary Table S2.

### Virulence factor analysis

Virulence gene analysis identified several genes associated with bacterial pathogenicity, with an average of 55 virulence genes per genome. All isolates harboured a core set of virulence genes, including ferric enterobactin transport (*fepC*), outer membrane protein (*ompA*), and enterobactin synthase component (*entB*). Additional clinically relevant virulence genes included adhesins (*aggR*, *fimA-H*), toxin-associated genes (*est*, *aatA*), and iron acquisition systems (*entA-B*, *iucA-D*, *iroE*).

**Fig. 2:**
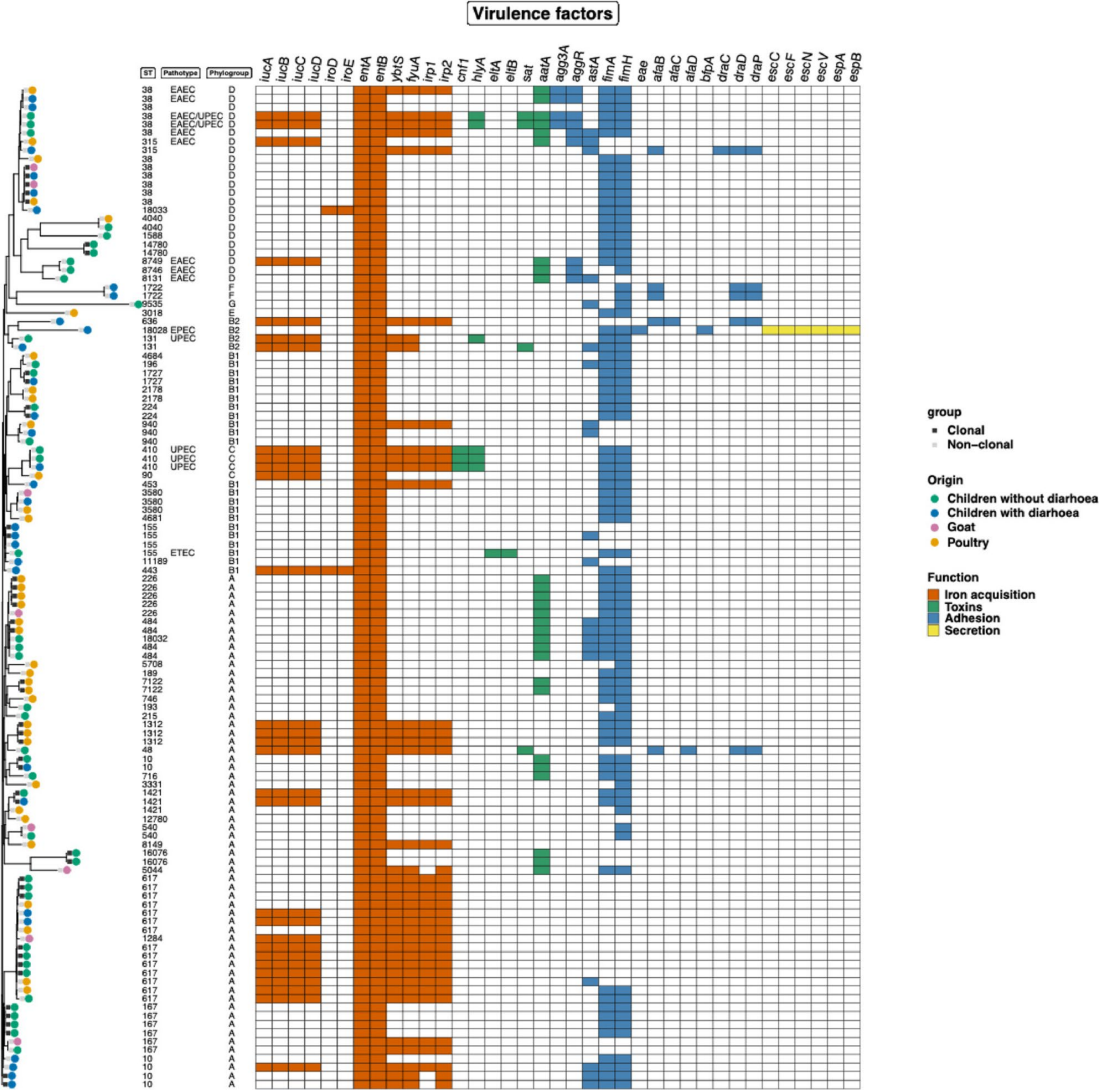
Heatmap illustrating the distribution of ESBL-producing *E. coli* virulence genes among isolates from various sample types. Note: Several additional virulence genes present in the genome are not depicted in the data, as only a selection of important virulence genes is shown for visibility reasons

Virulence genes associated with diarrheagenic *E. coli* pathotypes were detected in 11 isolates, including nine enteroaggregative *E. coli* (EAEC), one enterotoxigenic *E. coli* (ETEC), and one enteropathogenic *E. coli* (EPEC). Six isolates were classified as uropathogenic *E. coli* (UPEC). Among isolates from children with diarrhoea, nine were identified as EAEC, one as ETEC, and one as EPEC (Fig. 2).

Phylogenetic analysis showed that most EAEC isolates were associated with ST38, while three of the six UPEC isolates belonged to ST410 (Fig. 2). ST38 was identified in isolates from children, poultry, and goats. Overall, virulence-associated sequence types were distributed within dominant phylogroups and included isolates from multiple host sources.

## Discussion

The spread of AMR extends beyond human healthcare practices as it is intricately linked to the interactions between humans, animals, and the environment [20]. Therefore, this study assessed the molecular characterization of 117 ESBL-producing *E. coli* isolates from Ghanaian children (both with and without diarrhoea) and farm animals (poultry and goats).

### Genomic diversity of ESBL-producing *E. coli* in the study population

Phylogenetic analysis revealed substantial heterogeneity, with 49 STs identified among 117 ESBL-positive *E. coli* isolates after upload to EnteroBase, including three novel STs (ST18028, ST18032, and ST18033). The identification of three novel *E. coli* STs (ST18028, ST18032, and ST18033) expands the global MLST database and provides unique insights from a West African setting where genomic data remain limited. Additionally, the discovery of the new STs underscores the ongoing evolution of ESBL-producing *E. coli* in local reservoirs. Adding these lineages to international databases is essential for improving global surveillance. This will allow earlier recognition of region-specific clones that could spread more widely. The globally pandemic high-risk sequence type ST131 [19], frequently associated with *bla*_CTX−M−15_, was identified in two isolates in this study.

The bacterial strains in our study exhibited over 70 different AMR genes with high resistance to aminoglycosides, fluoroquinolones, tetracyclines, sulfonamides, and trimethoprim, fulfilling the “multidrug-resistance” definition as reported elsewhere [21]. Globally, *bla*_CTX−M−15_ is the most common ESBL mechanism in *E. coli* [22, 23], which aligns with our findings. However, none of these *bla*_CTX−M−15_ *E. coli* isolates carried other *bla*_CTX−M_ types, although they frequently coexisted with aminoglycoside resistance genes, tetracycline resistance genes, and fluoroquinolone resistance genes.

As expected, because gene expression and regulatory mechanisms influence phenotypic observation [24], genotypic and phenotypic susceptibility results did not entirely correspond (Table 2). Considering the time gap between phenotypic testing and genomic analysis, hypothetically interfering factors like plasmid loss in spite of appropriate sample storage and transport might have affected the genotype-phenotype correlation [25]. Additionally, the presence of resistance genes does not always translate into phenotypic resistance because of regulatory mutations, gene truncation, low expression levels, or gene silencing [26]. Equally, phenotypic resistance without identifiable genes may result from alternative mechanisms such as efflux pump overexpression, reduced membrane permeability, or chromosomal mutations [27]. Chromosomal mutations, particularly in *gyrA* and *parC* **(**Fig. 1**)**, may likely contribute to fluoroquinolone resistance independently of plasmid-mediated genes. This highlights the importance of considering both chromosomal and plasmid-mediated mechanisms when interpreting molecular AMR profiles.

The study detection of EPEC-, EAEC-, and ETEC-related virulence genes in 11 ESBL-producing *E. coli* isolates is consistent with findings from healthy human and livestock studies in Ethiopia [28] and across Europe [29]. UPEC-associated genes identified in six isolates may highlight a possible role in recurrent UTIs [30, 31]. In addition, eight phylogenetic groups were identified, with phylogroup A predominating, followed by D and B1, consistent with previous findings in Ghana [32]. The dominance of phylogroup A suggests many strains are commensal or environmental, which often carry fewer virulence factors but can serve as reservoirs for AMR genes. However, the presence of phylogroups D and B1 indicates circulation of potentially pathogenic extraintestinal *E. coli* (ExPEC) associated with urinary tract and bloodstream infections [33]. Our results on MGEs also align with a previous study conducted in Ghana [11], suggesting a continued circulation within the region.

### Overlapping sequence types and resistance determinants between human and animal isolates

The observations in the study align with a study from Ghana, which documented a similar diversity of STs among human and poultry isolates [10] and may reflect shared reservoirs. This is likely facilitated by the close proximity of the living conditions of both the children and the animals. Notably, ST10 was identified in children and farm animals in our study, consistent with a previous report from Ghana [11] and in environmental samples from Nigeria [34], underlining its likely potential of spreading across species. The identification of globally recognized ESBL-associated STs such as ST617 and ST38 in both human and animal isolates is noteworthy due to their role in the spread of AMR. These STs have been implicated in the spread of multidrug resistance across both community and healthcare settings in various regions, including Africa [35, 36]. Thus, supporting the need for continued genomic surveillance within a One Health framework.

MGE families were evenly distributed between children and poultry isolates. IncF-type MGEs have been early described as potent drivers of the dissemination of ESBL-type resistance in Enterobacterales, and are known to mediate resistance to third-generation cephalosporins and even carbapenems [37–40]. However, as replicon typing just indicates potential plasmid families, it does not confirm identical plasmids or transmission events. Potential circulation across shared reservoirs remains speculative and cannot be concluded just based on the data provided.

### Public health implications for antimicrobial resistance surveillance in Ghana

The findings of new STs in the study are clinically and epidemiologically significant because some of the novel clones harbor resistance (*bla*_CTX−M−15_) or virulence gene (*escC*) combinations. Such genetic profiles may alter their pathogenic potential and enhance their ability to persist within different hosts and environments. The study’s high frequency of *bla*_CTX−M−15_ gene isolates has significant implications for clinical treatment. This is particularly concerning in low-resource settings, where access to new antibiotics is limited [7]. Multidrug-resistant profiles are a challenge for empirical therapy and increase the risk of treatment failure, prolonged illness, and higher healthcare costs [4, 5]. This may lead to overstretching of the healthcare system as clinicians and veterinarians are forced to rely on last-resort antibiotics, which may be unaffordable or unavailable in many healthcare facilities in Ghana. STs across hosts within these phylogroups suggest ecologically versatile lineages circulating in a One Health context [36].

### Limitations of this study

The interpretation of the study results is limited by the regional focus as well as by the low number of included animal species and ESBL-positive *E coli*. Second, the time gap between phenotypic testing and genomic analysis might have affected differences in the phenotypic and genetic resistance testing results, possibly due to phenomena like plasmid loss during deep-frozen storage. Our plasmid analysis is based on replicon typing, and the observed similarities between host groups reflect shared plasmid types, but do not confirm identical plasmids. As a result, it was not possible to accurately determine with certainty whether similar or identical MGEs were truly shared across isolates. Because this study relied on short-read sequencing, it was not possible to reconstruct complete plasmid sequences; therefore, replicon similarity should not be interpreted as evidence of identical plasmids or plasmid transmission, and long-read sequencing would be required to confirm plasmid identity and transmission.

## Conclusions

Our study contributes to existing knowledge on the molecular epidemiology of ESBL-positive *E. coli* in Ghanaian children and farm animals. Thus, it represents one of the first WGS-based analyses of ESBL-positive *E. coli* from children and farm animals in the Agogo community. By integrating genomic data from multiple host sources, we provide novel insights into the overlapping genetic diversity, resistance determinants and high-risk clones circulating locally. These replicon types suggest shared reservoirs or overlapping ecological niches. Importantly, three novel sequence types (ST18028, ST18032, ST18033), which are region-specific clones, were detected, expanding the global MLST database with unique genomic data from Ghana, a region where such information remains scarce. This region-specific genomic evidence fills an important knowledge gap and establishes a baseline for future molecular epidemiological surveillance and One Health–focused investigations.

## Supplementary Information

Supplementary Material 1.

Supplementary Material 2.

Supplementary Material 3.

Supplementary Material 4.

Supplementary Material 5.

Supplementary Material 6.

## Data Availability

The raw sequence data reported in this paper have been deposited in the Genome Sequence Archive, accessible at (https:/ngdc.cncb.ac.cn/gsa) with the following accession code: CRA023281 or https://ngdc.cncb.ac.cn/gsa/browse/CRA023281.
