# Supplementary material for: Molecular characterization of extended-spectrum beta-lactamase-producing Escherichia coli among children and farm animals in Agogo, Ghana
Source: BMC Microbiol. 2026 Mar 25;26:429. doi: 10.1186/s12866-026-04978-w (PMC13137593; doi:10.1186/s12866-026-04978-w)
Supplement: Supplementary file 1 — Supplementary Material 1. [file 12866_2026_4978_MOESM1_ESM.pdf]

KWAME NKRUMAH UNIVERSITY OF SCIENCE AND TECHNOLOGY
COLLEGE OF HEALTH SCIENCES
SCHOOL OF MEDICAL SCIENCES / KOMFO ANOKYE TEACHING HOSPITAL
COMMITTEE ON HUMAN RESEARCH, PUBLICATION AND ETHICS

Our Ref: CHRPE/AP/674/19

4th December, 2019.

Dr. Denise Dekker
Bernhard-Nocht Str.
74, D-20359 Hamburg
GERMANY

Dear Sir,

LETTER OF APPROVAL

Protocol Title Renewal:: ***"Genetic Adaptation of Non-Typhoid Salmonella within Human and Animal Reservoirs in sub-Sahara Africa."***

Proposed Site: ***Kwame Nkrumah University of Science and Technology (KNUST) Kumasi, Ghana; Agogo Presbyterian Hospital, Asante Akim North; Kumasi Centre for Collaborative Research in Tropical Medicine (KCCR), Kumasi, Ghana and Bernhard Nocht Institute for Tropical Medicine (BNITM), Hamburg, Germany.***

Sponsor: ***Deutsche Forschungsgemeischaft (DFG), Bonn, Germany.***

Your submission to the Committee on Human Research, Publication and Ethics on renewal to protocol No. CHRPE/AP/593/18 dated 26th October, 2018 refers.

The following documents were reviewed by the Committee:

- A notification letter of 30th August 2017 from the Agogo Presbyterian Hospital (study site) indicating approval for the conduct of the study in the Hospital.
- A Completed CHRPE Application Form.
- Participant Information Leaflet and Consent Form.
- Research Protocol.
- Case Report Forms.

The Committee has considered the ethical merit of your proposed renewal and approved it. The approval is for a fixed period of one year, beginning 20th December, 2019 to 19th December, 2020 renewable thereafter. The Committee may however, suspend or withdraw ethical approval at any time if your study is found to contravene the approved protocol.

Data gathered for the study should be used for the approved purposes only. Permission should be sought from the Committee if any amendment to the protocol or use, other than submitted, is made of your research data.

The Committee expects a report on your study annually or at the close of the project, whichever one comes first. It should also be informed of any publication arising from the study.

Yours faithfully,

Osomfo Prof. Sir J. W. Acheampong MD, FWACP
Chairman
