# Supplementary material for: Molecular characterization of extended-spectrum beta-lactamase-producing Escherichia coli among children and farm animals in Agogo, Ghana
Source: BMC Microbiol. 2026 Mar 25;26:429. doi: 10.1186/s12866-026-04978-w (PMC13137593; doi:10.1186/s12866-026-04978-w)
Supplement: Supplementary file 2 — Supplementary Material 2. [file 12866_2026_4978_MOESM2_ESM.docx]

**Information Sheet on the Informed Consent-stool sample**

**Participant ID:______________________**

1. **Who are we?**

We are a research group including doctors, nurses, laboratory technicians and other medical staff who work at the National Institute For Medical Research (NIMR), and the Bernhard Nocht Institute for Tropical Medicine in Germany.

The responsible researchers are:

Dr Denise Dekker from the Bernhard Nocht Institute for Tropical Medicine (BNITM) in Hamburg, Germany.

Prof Dr John Lusingu from the National Institute for Medical Research (NIMR) in Tanga, Tanzania.

1. **What are we trying to find out?**

We are trying to find out whether your child or yourself carry a certain organism, named “*Salmonella*”, in the gut. This organism can cause diarrhoea or severe invasive disease if the bacterium enters your bloodstream. When studying this organism, we want to find out, whether you got this organism. At the same time we test animals such as farm and community animals but also meat for Salmonella. This way we can compare the *Salmonella* collected from humans and animals and see if they are the same or if they are different. Once we know more about this organism, we can develop better ways to protect your child from getting sick with this organism. We will also look for other bacterial pathogens that can cause your child having diarrhoea such as *Campylobacter*, *Arcobacter, Shigella* and diarrhoeagenic *Escheria coli*. As for *Salmonella*, once we know what bacteria your child has we will be able to prescribe the appropriate treatment.

1. **How are we going to do this?**

Your child will be asked to provide a small stool sample. This sample will be analysed, to see whether “*Salmonella*” and other bacteria can be found in the gut.

We will ask you some questions to your personal life and document these on a piece of paper. These questions will be for example, how many children you have, or how you are cooking or what kind of education you have.

After you have given the stool sample of your child to our study staff; tests will be performed in the local laboratory and overseas at the BNITM in Germany or, if necessary, in another laboratory. At the local laboratory and the BNITM the samples will be stored for 10 years and in this way will be available for further analysis, if necessary. Your stool sample will not be stored.

1. **What means that my child’s participates voluntarily?**

The participation your child in this study is voluntary. This means that you do not have to enrol your child in the study, if you do not want to. You can withdraw your child from the study at any time, without having to give a reason and without any penalty. If you chose to withdraw your child, your child will continue to receive the hospitals standard diagnosis and standard treatment.

1. **What kind of treatment will my child receive?**

In case your child is sick with “*Salmonella*” or any of the other bacteria and has diarrhoea, he/she will receive the appropriate treatment, which consists usually of rehydration therapy. The study doctor and the nurse will provide the treatment and if necessary further treatment will take place according to the usual national recommendations or the Hospital’s treatment protocols. In case you chose to withdraw your child, your child will receive the standard treatment according to existing national guidelines.

1. **What will happen with my private information?**

All private information including for example your name, your child’s name, birthdays, clinical information and test results will be treated confidentiality. This means that your records will get a number and names will not be openly traceable. All the results and the answers to our questions that you give us will be treated confidentially at all times and will not be made publically with the name of your child or your own name.

1. **Contact information for questions or concerns**
2. If you have any questions about this study, or if you want you/your child to stop being part of the study, please contact Dr John Lusingu, NIMR (Tel: 0787679515). You can also contact any of our study staff at the hospital.
3. If you have any questions about your rights as a study patient, or if you think **you or** your child **have** been injured because of this study, please contact the Chairman of the National Health Research Ethics Committee (NatHREC) on +255 222 121 400/390
