# Supplementary material for: Molecular characterization of extended-spectrum beta-lactamase-producing Escherichia coli among children and farm animals in Agogo, Ghana
Source: BMC Microbiol. 2026 Mar 25;26:429. doi: 10.1186/s12866-026-04978-w (PMC13137593; doi:10.1186/s12866-026-04978-w)
Supplement: Supplementary file 4 — Supplementary Material 4. [file 12866_2026_4978_MOESM4_ESM.docx]

Supplementary Material (SM1)

**Molecular characterization of extended-spectrum beta-lactamase-producing *Escherichia coli* among children and farm animals in Agogo, Ghana**

(SM1.1) (https://github.com/tseemann/shovill) for assembly, reference search using MASH v2.2.2,

(SM1.2) (https://github.com/ablab/quast).

(SM1.3) (https://github.com/OLC-Bioinformatics/ConFindr)

(SM1.4) (https://github.com/chklovski/CheckM2).

(SM1.5) (https://github.com/oschwengers/bakta).

(SM1.6) (https://github.com/gtonkinhill/panaroo)

(SM1.7) (https://mafft.cbrc.jp/alignment/software/).

(SM1.8) (https://gitlab.com/bfr_bioinformatics/chewieSnake).

(SM1.9) (<http://www.iqtree.org>).

(SM1.10) (<https://github.com/ncbi/amr>)

(SM1.11) (https://github.com/tseemann/abricate),

(SM1.12) (https://github.com/tseemann/abricate)

(SM1.13) (https://github.com/phac-nml/mob-suite)

(SM 1.14) (<https://github.com/tseemann/mlst>))
