# Supplementary material for: Molecular characterization of extended-spectrum beta-lactamase-producing Escherichia coli among children and farm animals in Agogo, Ghana
Source: BMC Microbiol. 2026 Mar 25;26:429. doi: 10.1186/s12866-026-04978-w (PMC13137593; doi:10.1186/s12866-026-04978-w)
Supplement: Supplementary file 5 — Supplementary Material 5. [file 12866_2026_4978_MOESM5_ESM.docx]

| **Supplementary Table 1: Distribution of Major Antimicrobial Resistance (AMR) Genes Among 117 *E. coli* Genomes** | | | |
| --- | --- | --- | --- |
| **Resistance Class** | **Gene** | **Function/Mechanism** | **Prevalence % (n/N)** |
| **β-lactams (ESBL & others)** | **blaCTX-M-15** | ESBL | 88% (103/117) |
|  | blaTEM-1 | Broad-spectrum β-lactam resistance | 41.9% (49/117) |
|  | blaOXA-1 | β-lactam resistance | 18.8% (22/117) |
| **Aminoglycosides** | aph3-Ib | Phosphotransferase | 59.0% (69/117) |
|  | aph6-Id | Phosphotransferase | 58.0% (68/117) |
|  | acc6-Ib-D181Y | Aminoglycoside-modifying enzyme | 17.9% (21/117) |
|  | aadA5 | Aminoglycoside adenyltransferase | 16.2% (19/117) |
|  | aadA1 | Aminoglycoside adenyltransferase | 12.0 (14/117) |
| **Sulphonamides** | sul1 | Dihydropteroate synthase variant | 27% (32/117) |
|  | sul2 | Dihydropteroate synthase variant | 57.3% (67/117) |
| **Quinolones** | qnrS1 | Plasmid-mediated quinolone resistance | 53.0% (62/117) |
|  | qepA4 | Efflux pump (quinolone resistance) | 7.7% (9/117) |
| **Tetracyclines** | tetA | Efflux pump | 52.1% (61/117) |
|  | tetB | Efflux pump | 32.5% (38/117) |
|  | tetD | Efflux pump | 5.13% (6/117) |
| **Macrolides** | mphA | Macrolide resistance enzyme | 30.8% (36/117) |
