# Supplementary material for: Molecular characterization of extended-spectrum beta-lactamase-producing Escherichia coli among children and farm animals in Agogo, Ghana
Source: BMC Microbiol. 2026 Mar 25;26:429. doi: 10.1186/s12866-026-04978-w (PMC13137593; doi:10.1186/s12866-026-04978-w)
Supplement: Supplementary file 6 — Supplementary Material 6. [file 12866_2026_4978_MOESM6_ESM.docx]

**Supplementary Table 2: Distribution of Mobile Genetic Elements (MGEs) Detected Across Isolates.**

| **MGE Type** | **Total Isolates (n)** | **Children with Diarrhoea** | **Children without Diarrhoea** | **Poultry** | **Goats** |
| --- | --- | --- | --- | --- | --- |
| *IncY* | 19 | 6 | 6 | 5 | 2 |
| *IncFIB* | 9 | 2 | 5 | 2 | 0 |
| *Col(BS512)* | 10 | 2 | 3 | 5 | 0 |
| *IncFII* | 6 | 2 | 4 | 0 | 0 |
| *rep_cluster_488* | 6 | 0 | 3 | 3 | 0 |
| *ColpVC* | 4 | 2 | 1 | 1 | 0 |
| *IncQ1* | 3 | 1 | 2 | 0 | 0 |
| *IncFIA* | 2 | 2 | 0 | 0 | 0 |
| *ColRNAI_rep_cluster_1987* | 2 | 1 | 0 | 1 | 0 |
| *Col(MG828)* | 1 | 0 | 1 | 0 | 0 |
| *IncHI1B* | 1 | 0 | 0 | 1 | 0 |
| *rep_cluster_1704* | 1 | 1 | 0 | 0 | 0 |
| *rep_cluster_2131* | 1 | 1 | 0 | 0 | 0 |
| *rep_cluster_2335* | 1 | 0 | 0 | 1 | 0 |
| *rep_cluster_2358* | 1 | 0 | 0 | 1 | 0 |
| *rep_cluster_2373* | 1 | 0 | 1 | 0 | 0 |
